# Hypoparathyroidism and pseudohypoparathyroidism in pregnancy: an Italian retrospective observational study

**DOI:** 10.1186/s13023-021-02053-3

**Published:** 2021-10-09

**Authors:** Gemma Marcucci, Paola Altieri, Salvatore Benvenga, Marta Bondanelli, Valentina Camozzi, Filomena Cetani, Luisella Cianferotti, Mirko Duradoni, Caterina Fossi, Ettore degli Uberti, Fausto Famà, Giovanna Mantovani, Claudio Marcocci, Laura Masi, Uberto Pagotto, Andrea Palermo, Simone Parri, Rosaria Maddalena Ruggeri, Maria Chiara Zatelli, Maria Luisa Brandi, On behalf of “Hypoparathyroidism Working Group” affiliated to the Italian Society of Endocrinology (S.I.E.)

**Affiliations:** 1 Bone Metabolic Diseases Unit, Department of Biomedical, Experimental and Clinical Sciences, University of Florence, Florence, Italy; 2 Endocrinology Unit and Prevention and Care of Diabetes, Alma Mater Studiorum University of Bologna, Bologna, Italy; 3 Department of Clinical and Experimental Medicine, University of Messina, Messina, Italy; 4 Department of Medical Sciences, Section of Endocrinology and Internal Medicine, University of Ferrara, Ferrara, Italy; 5 Endocrinology Unit, Department of Medicine (DIMED), University of Padua, Padua, Italy; 6 Department of Clinical and Experimental Medicine, University of Pisa, Pisa, Italy; 7 Department of Information Engineering, University of Florence, Florence, Italy; 8 Department of Medical Sciences, Section of Endocrinology and Internal Medicine, University of Ferrara, Ferrara, Italy; 9 Division of Endocrine and Minimally Invasive Surgery Department of Human Pathology in Adulthood and Childhood “G. Barresi”,, University Hospital “G. Martino” of Messina, Messina, Italy; 10 Endocrinology Unit, Fondazione IRCCS Ca’ Granda Ospedale Maggiore Policlinico, Department of Clinical Sciences and Community Health, University of Milan, Milan, Italy; 11 Unit of Endocrinology, Campus Bio-Medico University, Rome, Italy; 12 Department of Clinical and Experimental Medicine, Unit of Endocrinology, University of Messina, Messina, Italy

**Keywords:** Hypoparathyroidism, Pseudohypoparathyroidism, Hypocalcemia, Pregnancy, Breastfeeding, Treatment, Calcium carbonate, Calcitriol, Preterm birth, Miscarriages

## Abstract

**Background:**

Hypoparathyroidism (HypoPT) or pseudo-hypoparathyroidism (pseudo-HypoPT) during pregnancy may cause maternal and fetal/neonatal complications. In this regard, only a few case reports or case series of pregnant or lactating women have been published. The purpose of this study was to describe clinical and biochemical course, pharmacological management, and potential adverse events during pregnancy and post-partum in pregnant women with HypoPT or pseudo-HypoPT. This was a retrospective, observational, multicenter, study involving nine Italian referral centers for endocrine diseases affiliated with the Italian Society of Endocrinology and involved in “Hypoparathyroidism Working Group”.

**Results:**

This study identified a cohort of 28 women (followed between 2005 and 2018) with HypoPT (n = 25, 84% postsurgical, 16% idiopathic/autoimmune) and pseudo-HypoPT (n = 3). In HypoPT women, the mean calcium carbonate dose tended to increase gradually from the first to third trimester (+ 12.6%) in pregnancy. This average increase in the third trimester was significantly greater compared to the pre-pregnancy period (*p* value = 0.03). However, analyzing the individual cases, in 44% the mean calcium dosage remained unchanged throughout gestation. Mean calcitriol doses tended to increase during pregnancy, with a statistically significant increase between the third trimester and the pre-pregnancy period (*p* value = 0.02). Nevertheless, analyzing the individual cases, in the third trimester most women with HypoPT (64%) maintained the same dosage of calcitriol compared to the first trimester. Both mean calcium carbonate and calcitriol doses tended to decrease from the third trimester to the post-partum six months. Most identified women (~ 70%) did not display maternal complications and (~ 90%) maintained mean serum albumin-corrected total calcium levels within the low-to-mid normal reference range (8.5 ± 0.8 mg/dl) during pregnancy. The main complications related to pregnancy period included: preterm birth (n = 3 HypoPT women), and history of miscarriages (n = 6 HypoPT women and n = 2 pseudo-HypoPT women).

**Conclusion:**

This study shows that mean serum albumin-corrected total calcium levels were carefully monitored during pregnancy and post-pregnancy, with limited evaluation of other biochemical parameters, such as serum phosphate, 24 h urinary calcium, 25-OH vitamin D, and creatinine clearance. To avoid complications in mothers affected by (HypoPT) or (pseudo-HypoPT) and offspring, intense biochemical, clinical and pharmacological monitoring during pregnancy and breastfeeding is highly recommended.

## Background

Parathyroid hormone (PTH) is essential for calcium homeostasis, and pregnancy is known for an increased calcium requirement [1]. During pregnancy and breastfeeding, physiological changes of maternal calcium metabolism take place to ensure both an adequate fetal skeleton mineralization, with approximately 80% of the mineral accruing in the fetus in the third trimester [2], and a sufficient amount of calcium in the breast milk [3, 4]. Essential minerals, such as calcium, phosphate, and magnesium circulating in the pregnant woman are delivered against a concentration gradient by the placenta to the fetus [5, 6]. In case of low maternal serum calcium, to provide adequate amounts of calcium to the fetus, the ion is made available at the expense of the maternal skeleton [5–7].

A status of chronic maternal hypocalcemia, due to hypoparathyroidism (HypoPT) or pseudo-hypoparathyroidism (pseudo-HypoPT) not properly treated with calcium supplements and active vitamin D metabolites (calcitriol) or analogues (alfacalcidol), can cause a reduction in placental transfer of calcium to the fetus [4, 8–12]. However, fetal hypocalcemia does not develop until maternal serum calcium level is severely reduced [4]. In pregnancy, either maternal hypercalcemia or hypocalcemia may impact fetal development of the parathyroid glands [1, 4, 13, 14].

During pregnancy, the challenge of pharmacological management is to maintain normal calcium and phosphate levels in both plasma and urine, periodically evaluating these markers and the variable individual responses to treatment [15–18]. Calcium and active vitamin D requirements can change dramatically during pregnancy, which may require frequent dose adjustments [4, 15, 19]. The clinical course and therapeutic management of HypoPT during pregnancy is still not fully clarified, due to the few case reports or case series published in literature [4].

The aim of this study was to conduct a retrospective observational investigation on pregnant women affected by HypoPT or pseudo-HypoPT to evaluate clinical and biochemical course and pharmacological management during pregnancy and postpartum period.

## Subjects and methods

This is a large retrospective, observational, multicenter, study conducted in women affected by HypoPT or pseudo-HypoPT, who had at least one pregnancy. This project involved nine Italian referral centers for endocrine diseases, affiliated with the Italian Society of Endocrinology (Società Italiana di Endocrinologia, S.I.E.) and involved in the "HypoparaNet" research group (HypoPT working group) [20, 21].

The purpose of the project was to retrospectively review medical records evaluating the clinical and biochemical course and pharmacological management of women affected by chronic HypoPT or pseudo-HypoPT during pregnancy and the postpartum period, from 2005 to 2018.

The inclusion criteria included: (1) history of chronic HypoPT for ≥ 12 months post-diagnosis; or pseudo-HypoPT diagnosis; (2) history of one pregnancy, followed at specialized endocrinology centers; and (3) capability of providing written informed consent.

All clinical data were collected anonymously with the initials of the name and date of birth of each patient, using an electronic Excel sheet. The blood-urinary exams, collected on average every 4 weeks during pregnancy, were analysed and their average concentrations were calculated within each trimester of pregnancy. The calcium carbonate dosage is expressed in terms of elemental calcium.

Analysis of frequencies and descriptive statistics were performed using the IBM Statistical Package for Social Sciences (SPSS 20.0) for Windows (IBM, Armonk, NY, USA). Data are presented as mean ± SD (Standard Deviation), unless otherwise stated. Repeated measures-related differences were evaluated by using Student’s t-test for paired sample. P value of less than or equal to 0.05 was considered statistically significant. For all the variables that did not meet the assumptions for parametric analysis, the Wilcoxon Signed-Rank Test was employed to assess paired data. Due to sample size constraints, inferential analyses for repeated measures were performed only on the chronic HypoPT sample.

The study was approved by the Institutional Review Board (Comitato Etico Area Vasta Centro, AUOC, Florence, Italy) [number: 10641_oss; 16 May 2017]. Informed consent was collected in accordance with General Authorization to Process Personal Data for Scientific Research Purposes (Authorization no. 9/2013, The Italian Data Protection Authority). The study was conducted according to the Declaration of Helsinki.

## Results

### Study population

Twenty-five women with chronic HypoPT and 3 with pseudo-HypoPT, who had at least one pregnancy, were identified. The results presented are focused on the first pregnancies, since the analysis conducted in the small subgroup (n = 8) with second and third pregnancies showed no differences.

Most of the chronic HypoPT cases were post-neck surgical for thyroid goiter or tumor, followed by 2 probable autoimmune HypoPT cases (no *AIRE* gene mutations were detected, but one patient also had chronic autoimmune thyroiditis and another had celiac disease), and 2 idiopathic HypoPT cases. Two women with pseudo-HypoPT type 1B had *GNAS* gene (Guanine Nucleotide binding protein, Alpha Stimulating) methylation alterations, and 1 woman with pseudo-HypoPT type 1A had *GNAS* gene mutation (GNAS c.568_571del).

All data collected during pre-pregnancy period (previous six months) are summarized in Table 1. History of spontaneous miscarriages was present in 24% (6/25) women with HypoPT, and in 66.6% (2/3) women with pseudo-HypoPT. Among these, all the women had spontaneous miscarriages in the first trimester, and all except one had a single abortion. The only patient, who reported two spontaneous abortions, was also affected by thalassemia major in addition to post-surgical HypoPT. In 2 cases of spontaneous miscarriages, calcium therapy was stopped without consulting their physician and reported hypocalcemia.

**Table 1:** Descriptive data of women with chronic HypoPT and pseudo-HypoPT at the pre-pregnancy period (previous six months), regarding pharmacological treatment, biochemical exams, clinical manifestations, and other data

	Hypo-PT (n = 25)			Pseudo-HypoPT (n = 3)		
Medications	Number	Mean ± SD	Range	Number	Mean ± SD	Range
Calcium carbonate, mg/day	24	1.416.7 ± 876.5	500–4000	3	500	–
Calcium citrate, mg/day	1	500	–	0	0	–
Calcitriol, µg/day	25	0.79 ± 0.51	0.25–3.0	3	0.67 ± 0.14	0.50–0.75
Alfacalcidol, µg/day	1	0.25	0.25	0	0	–
Cholecalciferol, IU/day	9	977.5 ± 413.4	400–1600	1	1,600	–

Biochemistry^a^						
Albumin-corrected calcium, mg/dl [8.5–10.1]	15	**8.2** ± 0.7	6.8–9.1	2	9.3 ± 0.7	8.8–9.8
Total calcium serum, mg/dl [8.5–10.1]	25	**8.3** ± 0.8	6.7–9.6	3	9.1 ± 0.3	8.8–9.3
Phosphate serum, mg/dl [2.5–4.9]	19	4.3 ± 1.3	0.9–6.4	3	4.6 ± 0.7	3.8–5.1
Urinary calcium, mg/24 h [100–300]	10	201.3 ± 87.2	68–353.8	3	122 ± 96.3	36.0–226
Creatinine, mg/dl [0.5–1.3]	16	0.7 ± 0.1	0.5–0.9	3	0.7 ± 0.1	0.6–0.8
Creatinine Clearance, ml/min [> 60]	12	98.6 ± 16.0	76.6–120	3	76.3 ± 11.8	69.0–90
PTH, pg/ml [10–65]	24	**8.5** ± 4.1	1–18	3	40.3 ± 23.7	14.0–59.9
25 (OH) vitamin D, ng/ml [30–100]	23	**28.9** ± 11.1	13.4–50.2	3	**16.5** ± 8.8	8.5–26
Magnesium, mg/dl [1.5–2.6]	12	1.8 ± 0.4	0.8–2.2	3	1.9 ± 0.3	1.7–2.2

Miscellanea						
Average duration of chronic HypoPT before the first pregnancy	25	4.6 ± 3.6	1–15	–	–	–
Average age at first pregnancy	25	32.3 ± 5.3	21–45	3	33.6 ± 6	28–40
Mean dietary calcium intake, mg/daily	7	800 ± 230.9	500–1100	–	–	–
Body weight (Kg)	25	63.9 ± 14.0	43–100	3	62 ± 7.2	56–70

Hypo-PT or Pseudo-HypoPT-related Clinical Manifestations^b^	Number (Denominator)	Number (Numerator)	Frequency	Number (Denominator)	Number (Numerator)	Frequency
Paresthesia	25	13	52%	3	1	33.3%
Cramps/tetany	25	3	12%	3	0	0
Kidney stones	25	1	4%	3	1	33.3%
Extraskeletal calcifications	25	0	0	3	2	66.7%

^a^The reference ranges are given in brackets. Values outside of the reference range are typed **boldface**

^b^Nephrocalcinosis and kidney failure (creatinine clearance < 30 ml/min) are not listed because absent in all women

Only 3 women with HypoPT (12%, 3/25), average age 32 years (range: 29–35), underwent assisted reproduction treatment; in 1/3, the reason was infertility of the partner. Most of the women with HypoPT had a term birth (80%, 20/25); only 2 women (8%) had a post-term birth; 3 (12%) women had a preterm birth. All pseudo-HypoPT women had a term birth.

During pregnancy, 8/25 women with HypoPT (32%) reported maternal complications, including pre-eclampsia (n = 2), premature rupture of membranes (n = 2), polyhydramnios (n = 1), placenta previa (n = 1), and gestational diabetes (n = 3). One woman had gestational diabetes and pre-eclampsia during a triplet pregnancy.

One case of interventricular septal defect was reported as neonatal complication among the newborns of women with HypoPT, whereas 1 case of transient hypocalcemia and 1 case of respiratory distress with pulmonary hypertension were described in 2 newborns of women with pseudo-HypoPT, the second carrying the same mutation as his mother. However, complete data on the neonatological evaluation of newborns in all patients are not available.

Mean calcium dietary intake during pregnancy was 738.9 mg/day ± 211.8 (range: 500–1200), and during breastfeeding was 820 mg/day ± 268 (range: 600–1200); however, this data was available only for 10/25 (40%) women with HypoPT.

### Calcium and vitamin D supplementations

**Fig. 1:**
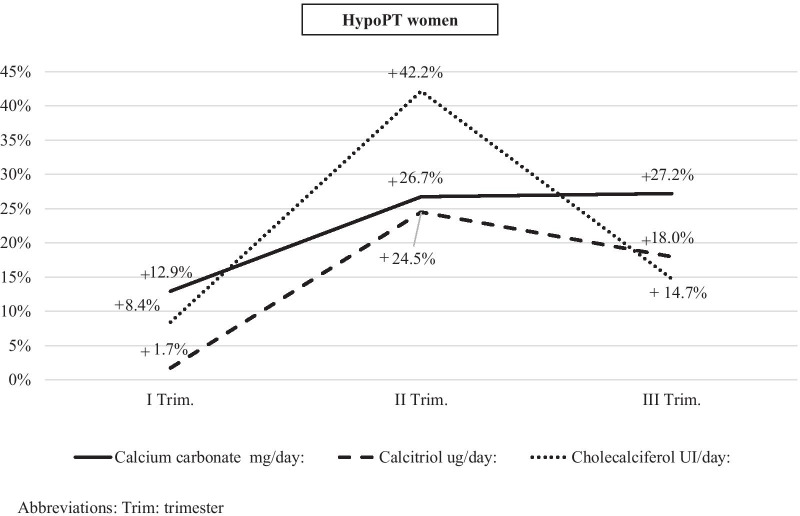
This figure shows the percentage differences of mean dosage of calcium carbonate, calcitriol and cholecalciferol during pregnancy compared to the pre-pregnancy semester in HypoPT women

In the group of women with HypoPT, the mean calcium carbonate dose tended to increase gradually from the first trimester to the third trimester (+ 12.6%) in pregnancy (Table 2). This average increase in the third trimester was significantly greater compared to the pre-pregnancy period (+ 385.5 mg/day, + 27.2%; *Z* value = −2.21, *p* value = 0.03) (Fig. 1).

**Table 2:** This table shows the supplementation with calcium and vitamin D (analogues and metabolites) and biochemical exams during three trimesters of the pregnancy in women with HypoPT and with Pseudo-HypoPT

				HypoPT women					
	I° trimester			II° trimester			III° trimester		
Medications	N	Mean ± SD	Range	N	Mean ± SD	Range	N	Mean ± SD	Range
Calcium carbonate, mg/day	25	1600 ± 1050	500–4000	25	1795 ± 1265.6	250–6000	25	1802 ± 1242	500–6000
Calcitriol, µg/day	25	0.8 ± 0.4	0–2	25	1 ± 0.7	0–3	25	0.94 ± 0.62	0–3
Cholecalciferol, IU/day	10	1059.7 ± 522.4	400–2000	8	1389.6 ± 1063.6	400–3666.7	8	1121 ± 653	400–2000

Other biochemical exams^a^									
Phosphate serum, mg/dl [2.5–4.9]	15	4.3 ± 1.2	1.1–6.3	17	4.2 ± 1.2	1.3–5.8	15	4.3 ± 1.1	1.8–5.8
Urinary calcium, mg/24 h [100–300]	5	231.9 ± 119	69–350	5	**301** ± 224.4	166–698	5	**37**9 ± 168	260–570
Creatinine, mg/dl [0.5–1.3]	15	0.7 ± 0.1	0.5–0.9	9	0.6 ± 0.1	0.4–0.8	12	0.7 ± 0.1	0.5–0.8
Creatinine Clearance, ml/min [> 60]	7	104.9 ± 19.3	75–123	5	110.6 ± 16.1	90–127	5	97.4 ± 13	90–120
25 (OH) vitamin D, ng/ml [30–100]	9	31.9 ± 10.3	13–44	7	38.5 ± 19.9	15–79.7	4	**21.5** ± 13.3	7–36.1
Magnesium, mg/dl [1.5–2.6]	8	1.7 ± 0.4	0.7–2	5	1.5 ± 0.4	0.7–1.9	3	1.9 ± 0.1	1.8– 1.9

				Pseudo-HypoPT women					
	I° trimester			II° trimester			III° trimester		
Medications	N	Mean ± SD	Range	N	Mean ± SD	Range	N	Mean ± SD	Range
Calcium carbonate, mg/day	3	875 ± 530	500–1250	3	875 ± 530	500–1250	3	1125 ± 177	1000–1250
Calcitriol, µg/day	3	0.7 ± 10	50–80	3	0.7 ± 10	50–80	3	0.8 ± 29	50–100
Cholecalciferol, IU/day	2	2466 ± 1225	1600–1333	2	2466 ± 1225	1600–3333	2	2466 ± 1225	1600–3333

Other biochemical exams^a^									
Albumin-corrected calcium, mg/dl [8.5–10.1]	3	9.3 ± 0.8	8.7–9.8	3	9.2 ± 0.2	9–9.4	3	9.1 ± 0.7	8.6–9.6
Phosphate serum, mg/dl [2.5–4.9]	2	4.1 ± 0.4	3.8–4.4	3	4.0 ± 0.7	3.5–4.8	3	3.9 ± 0.1	3.8–4
Urinary calcium, mg/24 h [100–300]	1	86 ± 0	86–86	1	215 ± 0	215–215	1	156 ± 0	156–156
Creatinine, mg/dl [0.5–1.3]	2	0.7 ± 0.1	0.6–0.7	2	0.6 ± 0	0.6–0.6	2	0.5 ± 0	0.5–0.5

^a^The reference ranges are given in brackets. Values outside of the reference range are typed **boldface**

However, analyzing the individual cases, in a subgroup of women (44%, 11/25) the mean calcium dosage remained unchanged throughout gestation (1222 ± 728 mg/day), as the mean dosage of calcitriol (0.75 ± 0.36 µg/day).

In 36% of the women (9/25), the mean calcium dosage increased by 844 mg/day from the first trimester (1565 ± 11.124) to the third trimester (2410 ± 1595). Only 12% women (3/25) showed a decrease by an average of 450 mg/day in the third trimester (2367 ± 777), compared to the first trimester (3000 ± 1000), without substantial variations in calcitriol dose (0.83 ± 0.14 µg/day).

The group of women who increased the calcium supplementation dose during pregnancy had a mean calcium dietary intake during pregnancy less than 1000 mg/day, and reported more episodes of tetany/cramps and/or paresthesia (77.7%, 7/9) associated to lower mean albumin-corrected total calcium levels (8.10 mg/dl) compared to those who could decrease the dosage (mean serum calcium levels: 8.73 mg/dl), or who kept the same dosage for the whole pregnancy (mean serum calcium levels: 8.9 mg/dl).

The average dosage of calcitriol taken by HypoPT women in the first trimester was equal to 0.8 µg/day. The latter tended to increase during the second trimester to 1 µg/day, and then slightly decrease in the third trimester to 0.94 µg/day, with an average increase of 0.13 µg/day (+ 16%) between the first and third trimester, and 0.14 µg/day (+ 18%) between the pre-pregnancy period and third trimester, with statistically significant differences (respectively *Z* value = −2.20, *p* value = 0.03 and *Z* value = −2.49, *p* value = 0.02). Analyzing the individual cases, in the third trimester, most women with HypoPT (64%, 16/25) maintained the same dosage of calcitriol compared to the first trimester, while only one patient (4.2%) decreased the dosage (− 0.25 µg/day), and 7 patients (28%) increased it (+ 0.50 µg/day).

Cholecalciferol supplementation was taken by 8/25 patients with HypoPT during pregnancy (mean dose: 1190 IU/day), with an average increase of 5.8% in the third trimester compared to the first trimester, and of 14.3% compared to the pre-pregnancy period. Magnesium supplementation was reported for only 3 patients with HypoPT during pregnancy.

The mean requirement of calcium and calcitriol supplementation tended to decrease from the third trimester to the post-partum six months by 445.6 mg/day (− 24.7%) and 0.1 mcg/day (− 11.1%), respectively, and compared to breastfeeding period by 265 mg/day and 0.1 mcg/day (Table 3). The average period of breastfeeding was 6.6 months ± 3.8 (range: 1–12; 16/25 HypoPT women). Subsequently, in the first post-lactation control, the mean requirement of calcium and calcitriol supplementation decreased, compared to the third trimester of pregnancy, by 310.4 mg/day (− 17%; *Z* value = −2.34, *p* value = 0.02) and 0.1 µg/day (− 12%), respectively, returning to doses similar to those taken during the pre-pregnancy period (Table 3).

**Table 3:** This table shows the supplementation with calcium and vitamin D (analogues and metabolites) and biochemical exams during six months post-pregnancy, breastfeeding period and I° control post-breastfeeding in women with HypoPT

	Six months post-pregnancy			Breastfeeding period			I° control post-breastfeeding		
Medications	N	Mean ± SD	Range	N	Mean ± SD	Range	N	Mean ± SD	Range
Calcium carbonate, mg/day	25	1356 ± 706.8	500–3000	16	1530 ± 726	500–3000	16	1491 ± 681.5	500–2600
Calcitriol, µg/day	25	0.8 ± 0.6	0.25–3	16	0.84 ± 0.63	0.25–3	16	0.82 ± 0.53	0.25–2.50
Cholecalciferol, IU/day	4	862 ± 275	600–1250	5	850 ± 275	600–1250	5	800 ± 200	600–1000

Biochemistry^a^									
Albumin-corrected calcium, mg/dl [8.5–10.1]	25	8.5 ± 0.7	7.4–9.4	16	8.5 ± 0.6	7.5–9.3	16	8.5 ± 0.5	7.7– 9.3
Phosphate serum, mg/dl [2.5–4.9]	12	**5** ± 0.8	3.8–6.6	10	**5** ± 0.84	3–5.2	10	4.9 ± 0.3	4.7–5.5
Urinary calcium, mg/24 h [100–300]	7	196 ± 80	90–304	6	198 ± 72.6	90–304	6	292.8 ± 71	240–397
Creatinine, mg/dl [0.5–1.3]	8	0.7 ± 0.2	0.7–0.9	7	0.7 ± 0.1	0.7–0.9	7	0.7 ± 0.1	0.68– 0.86
Creatinine Clearance, ml/min [> 60]	8	104 ± 10	87–118	7	106.8 ± 10.4	90–116	7	104.5 ± 10.3	90–114
25 (OH) vitamin D, ng/ml [30–100]	5	**24** ± 15.2	13–40	5	**24** ± 15.2	13–40	5	**25.8** ± 8.5	19–38

^a^The reference ranges are given in brackets. Values outside of the reference range are typed **boldface**

The group of women with pseudo-HypoPT confirmed a tendency to increase the average dosage required of calcium carbonate and calcitriol from pre-pregnancy period to pregnancy and from first to the third trimester (Table 2), as well as the trend in post-pregnancy (only one patient nursed for 6 months).

### Biochemical exams and clinical manifestations

**Fig. 2:**
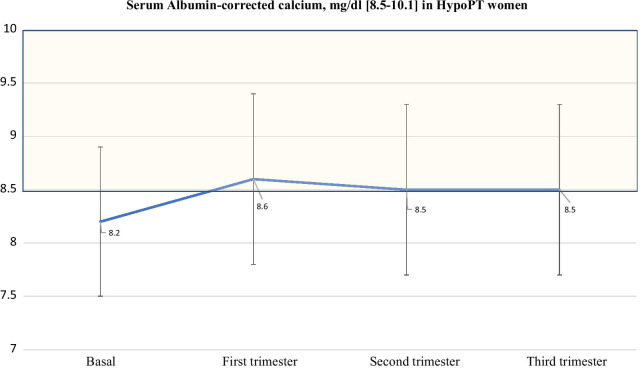
This graph shows the trend of mean values of serum Albumin-corrected calcium from the baseline (pre-pregnancy semester) to the third trimester. Bars are SDs, and shaded area identifies the normal range values of serum Albumin-corrected calcium levels

Mean serum albumin-corrected calcium levels remained approximately stable (8.5 ± 0.8 mg/dl; range: 6.9–9.8) throughout pregnancy (Fig. 2), without statistically significant differences with the pre-pregnancy period, post-pregnancy period (six months), breastfeeding period and post-lactation (Table 3). This pattern also applies to mean serum phosphate levels, except for an increasing trend in the six months post-pregnancy and in the period of breastfeeding. Only 2 cases with HypoPT showed average serum calcium levels steadily below 8 mg/dl despite treatment and increased doses of calcium carbonate during pregnancy, and another 2 cases reported hypercalcemia (11–13 mg/dl) during breastfeeding. Five cases with HypoPT reported average phosphatemia above the reference range during pregnancy (5/25) and breastfeeding (5/16).

The mean levels of 24-h urine calcium were measured only in 5/25 women with HypoPT during pregnancy, showing an increasing trend from the first trimester to third trimester (Table 2). In the post-pregnancy period, the mean values of 24-h urine calcium returned to normal levels, as in the pre-pregnancy period. Mean 25-OH vitamin D levels, reported only in few cases, were above 30 ng/mL, except in the third trimester and post-pregnancy period when mean levels were approximately 20 ng/mL (Table 2).

**Fig. 3:**
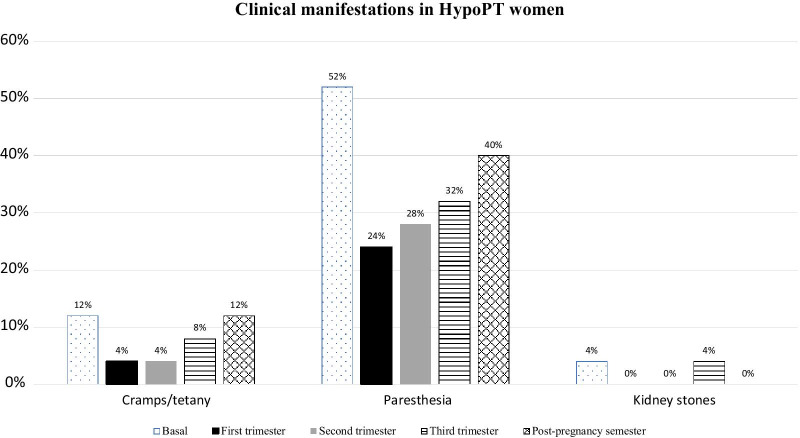
This graph shows the frequency (%) of HypoPT-related maternal clinical manifestations reported at baseline (pre-pregnancy semester), during pregnancy and post-pregnancy semester

On average, an increase in frequency of HypoPT-related clinical manifestations (Fig. 3) was reported in the third trimester compared to the first trimester, but the frequency was lower compared to the pre-pregnancy period, with a trend to increase again in the post pregnancy period.

In women with pseudo-HypoPT, the mean serum albumin-corrected calcium and phosphate levels were stable from pre-pregnancy through post-pregnancy, remaining within the normal range (Table 2). The mean levels of 24-h urine calcium were available only for one woman and were stably within the normal range from pre-pregnancy through post-pregnancy. Regarding clinical manifestations, no cramps/tetany were described; one woman had kidney stones, and two women reported episodes of paresthesia without differences in the frequency of episodes during the entire study period.

## Discussion

### Principal findings

This study included all cases (25 HypoPT women, 3 pseudo-HypoPT women) followed in pregnancy by 9 Italian referral centers for endocrine diseases, between 2005 and 2018. Most women of both groups had a natural pregnancy with a full-term birth, however the 24% of women with HypoPT reported a previous history of spontaneous miscarriages, 2 out of 3 women in the group of women with pseudo-HypoPT.

Most women of both groups increased significantly calcium supplementation and calcitriol dosage during the pregnancy compared to the pre-pregnancy period. The average calcium supplement doses were mostly maintained or increased throughout pregnancy, and the average calcitriol doses were mostly maintained at same dosage. During the breastfeeding in HypoPT women, the average dose of calcium supplements and calcitriol decreased significantly compared to the third trimester.

In most of cases the average serum albumin-corrected calcium levels were monitored and maintained in the low/normal reference range in the HypoPT women and in the normal reference range in the pseudo-HypoPT women throughout pregnancy and breastfeeding.

A general improvement in HypoPT-related clinical manifestations, such as cramps, tetany, and paresthesia, was shown during pregnancy compared to the previous six months.

### Results in the context of what is known

Until now, only limited case reports or case series of pregnant/lactating hypoparathyroid women have been published [1, 13, 15, 17, 22–26]. Among these, the largest case series described 12 cases with HypoPT [13, 26], while for women with pseudo-hypoPT, only case reports are available [27–30]. Therefore, this study identified the largest cohort of hypoparathyroid women, mostly with postsurgical HypoPT, which represents the prevalent form [31, 32].

In our study, in most cases pregnancy was natural and full-term. On the other hand, the few cases preterm delivery (3/25 HypoPT women) may be due to inadequately controlled serum calcium concentration (mean serum calcium levels < 8 mg/dl in the third trimester), which increases uterine irritability [4, 17, 33]. These 3 women also had maternal complications, such as gestational diabetes (n = 2), placenta previa (n = 1) and polyhydramnios (n = 1), that are unlikely to be related to HypoPT. A recent case series study reported four pregnancy (4/17) of HypoPT women (n = 12) complicated by polydramnios (24%), therefore further studies are needed but this potential complication could be considered in such patients [26].

Two patients with a previous history of spontaneous miscarriages reported stopping calcium therapy (without consulting their reference physician) and subsequent hypocalcemia. It is well known that an inadequate therapeutic management of HypoPT during pregnancy, with resulting poorly controlled serum calcium, can lead to a high risk of miscarriage [13, 14, 34, 35]. Regarding other miscarriages, no specific causes were reported. All the women, with the exception of one, reported an isolated abortion, therefore no poliabortivity was described in our analyzed sample. In this regard until today, there are no data published in the literature. In our patients, all the described abortions occurred in the first trimester, period in which the risk of spontaneous abortions is generally greater regardless of the HypoPT. Concomitant hypocalcemia with abortion was described in only two cases, however, probably also in the other cases there may have been fluctuations in serum calcium that may have contributed to abortions. The only patient, who reported two spontaneous abortions, was also affected by thalassemia major, therefore there was an overlap of two pathologies, and it is difficult to distinguish the role of the two pathologies in the etiopathogenesis of abortions.

Our study confirmed a wide variation in the required doses of calcium and calcitriol during pregnancy in hypoparathyroid mothers, as already described [4, 13, 26, 30, 36–40]. This variability may be due to variations in dietary calcium intake [4, 41, 42], which should be carefully monitored. This correlation could not be calculated in total group, due to the lack of data, however in more than half of patients, who increased the calcium supplementation dose, mean dietary intake of calcium during pregnancy was suboptimal. Moreover, these variations could also be due to the fact that the physiological hemodilution in pregnancy lowers the serum total calcium level, a reduction that may be interpreted as hypocalcemia [3, 14, 17, 38, 40, 41, 43].

This study confirmed the downtrend of calcium and calcitriol doses during the breastfeeding in women with HypoPT, as reported in other studies [3, 17, 19, 26, 38, 39, 41, 43, 44]. It is probably related to high levels of PTHrP [45] and could also be related to an increase in mean dietary intake of calcium during breastfeeding. Moreover, during the breastfeeding, hypercalcemia and hyperphosphatemia episodes were reported in literature [13, 17, 26], as described respectively in 2 and 5 of our HypoPT women. Therefore, a careful biochemical monitoring is also necessary in this period.

The analysis of biochemical exams showed mostly the maintenance of the average serum albumin-corrected calcium levels in the low/normal reference range throughout pregnancy. Indeed, biochemical monitoring of serum calcium levels was carried out constantly, as currently recommended by the guidelines [4, 15, 18]. However, serum/urinary calcium and phosphate oscillations were reported. A general improvement in HypoPT-related clinical manifestations during pregnancy could be attributed to the increase in synthesized calcitriol and PTHrp, as described above [4].

### Clinical implications

In a large retrospective population analysis, we show that serum calcium levels were strictly monitored during pregnancy and the post-pregnancy period, while other biochemical parameters, such as phosphate, 25-OH vitamin D, magnesium, and creatinine clearance tended to be poorly evaluated. Therefore, in future, also in light of the new guidelines, an accurate and complete biochemical monitoring is needed in pregnant/lactating hypoparathyroid women, in order to further reduce biochemical fluctuations and maternal/fetal complications [4, 15, 18]. Moreover, the average dietary calcium intake will have to be monitored more accurately, as it may be responsible for fluctuations in serum calcium. Cholecalciferol supplementation was reported only in about 30% of our women, a rate that the retrospective nature of the study could have underestimated. However, a greater attention in cholecalciferol supplementation should be carried out, as recommended by the guidelines [15].

### Research implications

Prospective investigations in women with HypoPT and pseudo-HypoPT during pregnancy and breastfeeding are necessary in order to increase knowledge about biochemical alterations, maternal and fetal clinical complications, and to improve the quality of care available today.

In particular, further studies are needed to confirm the upward trend of mean urinary calcium levels throughout gestation and then the return to levels similar to pre-pregnancy period during the first postpartum six months, described in our study in a small subgroup of patients with HypoPT as in other studies [4, 46]. In addition to the known physiological rise of calcitriol levels during pregnancy, especially in the third trimester, in hypoparathyroid women, the PTH deficiency and the conventional treatment with calcium and calcitriol could contribute to increased renal calcium excretion [4, 46–48], with associated increased risk of renal stones [4]. Further studies in hypoparathyroid women should be also conducted on phosphate homeostasis during pregnancy and especially during breastfeeding, period in which it tends to increase. It would be useful to measure the excretion of 24 h urinary phosphate and to study the effect of PTHrp. Regarding 25 OH vitamin D level, a slight decline of the circulating levels of 25-OH vitamin D in the third trimester, as described in our study, is congruent with the well-known increased conversion to calcitriol, in addition to the transfer of 25-OH vitamin D to the fetus [2, 4]*.* However, it has been analyzed in a few cases in our study, therefore further investigations are needed.

Regarding complications, further clinical investigations will be necessary to study maternal complications, such as reported in our study and in previous investigations [4, 17, 26, 33], in order to verify their incidence, relation with these disorders and to verify systematically neonatal complications through the support of colleagues specialized in neonatology.

### Strengths and limitations

The main strengths of this study are the inclusion of several cases of women with HypoPT with a multicentric investigation, and an extensive evaluation of biochemical exams, clinical complications and pharmacological management during pregnancy and post-pregnancy. On the other hand, missing data due to retrospective nature of the study represent the main study limitation.

## Conclusions

Our study confirms in a large cohort of women with HypoPT, that most women with HypoPT have an uncomplicated pregnancy (with the exception of those cases that receive undertreatment), while maintaining serum calcium concentrations within the low-to-mid normal reference range during the pregnancy period [4]. However, it highlights the risk of fluctuations in serum calcium and phosphate levels, maternal complications, and miscarriages. Therefore, the need for careful biochemical and clinical monitoring by endocrinologists and further studies to confirm the data described and evaluate maternal and fetal/neonatal complications are highlighted. In women with pseudo-HypoPT, unassisted and uneventful pregnancies have been reported in literature [18], but the cases described are very few, and close monitoring is essential, given the risk of complications.

## Data Availability

All data generated or analysed during this study are included in this published article.
